# Sleep quality and associated factors among nurses working at comprehensive specialized hospitals in Northwest, Ethiopia

**DOI:** 10.3389/fpsyt.2022.931588

**Published:** 2022-08-16

**Authors:** Tesfaye Segon, Habtamu Kerebih, Fanuel Gashawu, Bizuneh Tesfaye, Girum Nakie, Tamrat Anbesaw

**Affiliations:** ^1^Department of Psychiatry, College of Health Science, Mettu University, Mettu, Ethiopia; ^2^Department of Psychiatry, College of Medicine and Health Science, University of Gondar, Gondar, Ethiopia; ^3^Department of Psychiatry, College of Medicine and Health Science, Wollo University, Dessie, Ethiopia

**Keywords:** sleep quality, nurses, Gondar, Ethiopia, prevalence

## Abstract

**Background:**

Poor sleep quality is common among nurses. This problem possibly results in negative emotional and psychological consequences in nurses which secondary affect their work performances. However, in Ethiopia, there is a paucity of information about poor sleep quality and associated factors among nurses. This study aimed to assess the prevalence of poor sleep quality and associated factors among nurses working at comprehensive specialized hospitals in Northwest Ethiopia.

**Methods:**

An institutional-based cross-sectional study was conducted among 542 nurses who worked at University of Gondar (UOG), Tibebe Ghion, Felege Hiwot Comprehensive Specialized Hospitals, Ethiopia, who were incorporated into the study through a simple random sampling technique from 1 May to 2 June 2021. The Pittsburgh sleep quality index (PSQI) with a cut score of above 5 was used to assess sleep quality using a structured self-administered questionnaire. Other tools used are Depression Anxiety Stress Scales (DASS-21), Shift Work sleep disorders (SWSD), and Oslo-3 social support scales. Epi-Data version 3.1 was used for data entry and SPSS version 25 was used for data analysis. A multivariable logistic regression analysis was performed to identify variables that have a significant association with poor sleep quality among nurse professionals. The degree of association was assessed using an odds ratio (OR) with a 95% confidence interval (CI) at a two-tailed *p*-value of <0.05.

**Results:**

A total of 510 nurses were included in the study with a response rate of 94%. The study showed that the overall prevalence of poor sleep quality among nurses was 75.5% (95% CI (71.8, 79.1). Being female (AOR = 1.72:95% CI = 1.19, 2.28), depressive symptoms (AOR = 2.24:95% CI = 1.24, 3.85), anxiety symptoms (AOR = 2.12: 95% CI = 1.23, 3.62), stress (AOR = 2.85: 95% CI = 1.67, 4.82) and current alcohol drinking (AOR = 1.84 :95% CI = 1.27, 3.13) were significantly associated with poor sleep quality.

**Conclusion:**

The overall prevalence of poor sleep quality among nurses was high. Being female, depressive symptoms, anxiety symptoms, stress, and current alcohol drinking had been significantly associated with poor sleep quality. Therefore, it is essential to institute effective intervention strategies emphasizing contributing factors to poor sleep quality.

## Introduction

Sleep is an active cyclic biological phenomenon that is necessary for survival and one of the most significant human behaviors occupying roughly one-third of human life. It is important for the physiological process of the brain which comprises generally 8 h of nighttime sleep and 16 h of daytime wakefulness in humans (1). It is a state of reduced consciousness and responsiveness from which an individual can be aroused by external stimulus and which typically recurs for several hours every night (2).

Sleep quality is a measure of the feeling that a person would have of being energetic, active, and ready for a new day (3). Nurses are often scheduled to do healthcare activities during the day, evening, and night times. This results in abrupt deviations from the normal timing of sleep often disrupting their internal biological clock that leads to poor sleep quality that has an impact on their day-to-day functioning (4). This condition leads to a negative consequence of physiological, mental, and social aspects that could result from an early sign of underlying health problems (5). This impact leads to impairment in learning, focusing, safe functioning, and decision-making skills and increase the likelihood to make medical errors, work-related accidents or injuries, and absence from the workplace (6).

Sleep is increasingly becoming an important and growing global public health issue influencing millions of people globally (7). Poor sleep is a growing concern in the United States (US) with an estimated 50–70 million Americans suffering from sleep or wakefulness disorders indicating that poor sleep quality is a public health problem (8). A recent study indicated that the prevalence of poor sleep quality was higher in nurses than in the general population (9), with more than 50% of nurses reporting severe poor sleep quality (10), particularly those working night shifts (11). Worldwide, 61.0% of nursing staffs have poor sleep quality (12).

Various study results from different countries showed that the magnitude of poor sleep quality among nurses in the US 66% (13), the United Kingdom (UK) 78% (14), and Italy 54.6% (15). In another cross-sectional study from Asian countries, China hospitals 76.3% (16), and in South Korea 79.8% of nurses had poor sleep quality (17). In addition, in Africa, Nigeria 77.1% (18) and Ethiopia 70.6% (19) of nurses were affected by poor sleep quality. Many studies have shown a link between poor sleep quality and various risk factors among nurses such as being female (20–22), shift work (18–20), working in emergency departments (20), age (23), substance use like khat chewing (21, 22), having anxiety symptoms (24, 25), depressive symptoms (24), years of experience (21), and stress (19).

Nurses are the main workforce at hospital institutions responsible for direct and uninterrupted care to a patient with high pressure that is highly susceptible to occupational stress. If they are under such a stress state for a long time, then they may experience lassitude, anxiety symptoms, depressive symptoms, poor sleep quality, and other psychological problems (26). Despite the high magnitude of poor sleep quality among nurses and its negative emotional and physical consequences on the life of nurses and their function, little is known in African countries especially in Ethiopia about poor sleep quality and associated factors among nurses. The previous research also recommended assessing the association between sleep problems and depressive symptoms. So, in this study, we assessed by adding other variables such as depressive symptoms, anxiety symptoms, stress, and shiftwork sleep disorder (19). Therefore, this study aims to assess the prevalence of poor sleep quality and identify the factors associated among nurses working in comprehensive specialized hospitals. This could help as an input to make future interventions to improve nurses’ sleep quality. The future intervention will be further promoting their physical and psychological health as well as reduce workplace injury, enhance patient outcomes. The null hypothesis of the current study is that no statistically significant differences between poor sleep quality and profession among nurses would be expected.

## Materials and methods

### Study design, study area, and period

An institutional-based cross-sectional study design was employed from 1 May to 2 June 2021. The study was conducted in three comprehensive specialized hospitals (CSH) named “University of Gondar (UOG),” “Tibebe Ghion,” and “Felege Hiwot” Comprehensive Specialized Hospitals (CSH), which are located in the Northwest part of Ethiopia.

### Source population

All nurses working at UOG, Felege Hiwot, and Tibebe Ghion comprehensive specialized hospitals.

### Study population

Those who were available during the data collection period.

### Inclusion and exclusion criteria

All nurses working in the three hospitals were included, and nurses who were acutely ill during the data collection period at the hospitals were excluded.

### Sampling procedure and sampling techniques

#### Sample size determination

The sample size was estimated by using a single population proportion formula. The sample size was calculated with the following assumptions: n = initial sample size needed for this study α = confidence interval (CI) 95%, margin of error 4%, the prevalence (p) of poor sleep quality among nurses 70.6% which was taken from a similar study from Jimma, Ethiopia (19). The sample size with a z-value of 1.96 and marginal error of 4% sample was calculated as

$$n=\frac{{\left(z/2\right)}^{2}\,P\,\left(1-p\right)}{{\text{d}}^{2}}$$

Assumption: *n* = initial sample size need for this study α = confidence interval (95%) p = proportion of = 70.6% (0.706) (19) *d* = marginal error of 4% (z α/2) 2 = 1.96.

$$n=\frac{{\left(1.96\right)}^{2}\,0.706\,\left(0.294\right)}{{\left(0.04\right)}^{2}}=492$$

After adding 10% (50) non-response rate; finally, the total sample size was **542**.

#### Sampling procedure

**FIGURE 1:**
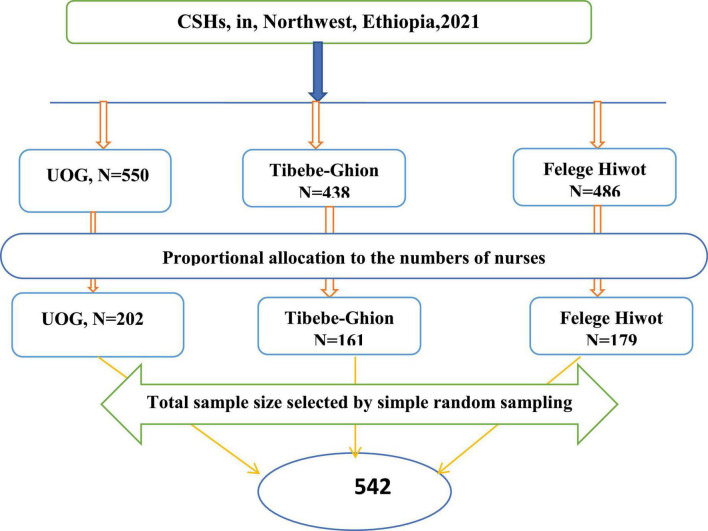
Proportional allocation of sample size among nurses working at comprehensive specialized hospitals in Northwest, Ethiopia, 2021 (*n* = 542).

The sample of the study was drawn from the three comprehensive specialized hospitals in North West, Amhara region, Ethiopia. The total calculated sample size (542 nurses) was proportionally allocated to each hospital based on the number of their nurses. A computer-generated simple random sampling method was employed to select nurses by using their sampling frame from each hospital (Figure 1).

### Variables

#### Dependent variable

Sleep quality: good/poor.

#### Independent variables

##### Socio-demographic factors

Sex, age, marital status, educational status, monthly income, working unit, and year of experience.

##### Psychological, shift work, and clinical characteristics

shift work sleep disorder, depressive symptoms, anxiety symptoms, stress, and comorbidity of medical illness (like asthma, cardiovascular, diabetes, hypertension, epilepsy, etc. …).

##### Psychosocial factors and behavioral characteristics

Social support, alcohol use, khat use, use of shisha (cannabis), and smoking cigarettes.

### Data collection tools

The data was collected by using a pretested, structured, self-administered questionnaire. Socio-demographic characteristics like; sex, age, marital status, educational status, monthly income, work unit, and year of experience.

An outcome variable (sleep quality) was assessed by using Pittsburgh Sleep Quality Index (PSQI) a self-report standard questionnaire item introduced in 1989 as an instrument to measure sleep quality (27). It consists of 19 questions evaluating the following 7 domains: subjective sleep quality, sleep latency, sleep duration, habitual sleep efficiency, sleep disturbances, sleep medication use, and daytime dysfunction. Each question has a response scale with scores ranging from 0 to 3 where zero refers to normal, one refers to mild dysfunction, two refers to moderate dysfunction, and three severe dysfunctions. A global subjective sleep quality score ranges between 0 and 21. It was validated in Ethiopia to measure sleep quality status in community settings and among adults with a sensitivity of 82% and specificity of 56.2% with Cronbach’s alpha of 0.81 (28). The Cronbach alpha of PSQI in the current study was 0.78. Using a global scale PSQ, consider having poor sleep quality, if the score is >5, whereas, good sleep if the score is = 5 (27).

Shift Work sleep disorders (SWSD) were assessed by three questions previously developed and used specifically to assess/diagnose SWSD in epidemiological studies. These questions adhere to the symptoms/criteria listed in the ICSD-3 with three yes/no questions. (1) Do you experience difficulties with sleeping or excessive sleepiness? (yes/no), (2). Is the sleep or sleepiness problem related to a work schedule where you have to work when you would normally sleep? (yes/no), (3). Has this sleep or sleepiness problem related to your work schedule persisted for at least 3 months? (Yes/no). If respondents had to answer “yes” to all three questions to fulfill the criteria for shift work sleep disorder (29).

Depression, anxiety, and stress were measured using Lovibond and Lovibond’s short version of the DASS-21. It is 21 items in three domains. Each domain comprises seven items assessing symptoms of depression, anxiety, and stress. Participants were asked to indicate the presence of symptoms in each domain over the past week scoring from 0 (did not apply at all) to 3 (applied most of the time). Scores from each dimension were summed. Finally, the values obtained were multiplied by 2. Cutoff scores of 10 and above, 8 and above, and 15 and above indicate a positive screen for the symptoms of depression, anxiety, and stress, respectively (30). The DASS-21 subscale reliability was with Cronbach’s alpha 0.75 for depressive symptoms, 0.79 for anxiety symptoms, and 0.73 for stress in a pre-tested questionnaire. Substance use was assessed by the ASSIST questionnaire which comprises substance use its assessment which is currently used and ever use were adapted from the ASSIST (Alcohol, Smoking, and Substance Involvement Screening Test), a well-validated instrument developed by the World Health Organization (WHO) was used (31). Current substance use, if they use at least one of a specific substance for non-medical purposes within the last 3 months (such as alcohol, khat, tobacco) (31). The Oslo 3 Social Support Scale was applied to know the level of social support of nurses. The scale divides the level of social support into three poor social support (3–8), moderate social support (3–14), and strong social support (12–14) (reliability of Cronbach’s α = 0:91 (32). History of known chronic medical illness was assessed by yes/no questions.

### Data collection procedure

Data were collected by three (for each hospital) trained BSc psychiatry professionals. The study used standardized and validated assessment tools. Each section of the questionnaire was prepared in English and then translated into the local language Amharic, and to ensure its understandability and consistency, then back-translated to English by an independent person. The training was provided to each hospital data collector for 1 day on assessment tools, how to collect data using the tools, methodology, ethical concerns, and how to supervise the data collection process. A pretest was done before the actual study on 5% (28) of the participants in Debre-Tabor Referral hospital to identify potential problems in data collection tools and modification of the questionnaire. Data collectors were supervised, and the filled questionnaires were checked daily. Each day throughout the data collection period, the completed questionnaires were assured of completeness and consistency. The collected data were entered into the computer, then checked and processed appropriately.

### Data processing and analysis

Data were coded, entered, and cleaned using Epi-Data version 3.1 and then exported to SPSS version 25 for analysis. Then, the data were analyzed to generate descriptive statistics: means, frequency, percentages, and standard deviations. Bivariate and multivariable logistic regression analysis was performed to identify associated factors to outcome variables. All variables with a *p*-value less than 0.25 in the bivariate analysis were entered into the multivariable logistic regression model. Multivariable logistic regression analysis was employed to control for possible confounding effects and to determine the presence of a statistically significant association between explanatory variables and outcome variables. The model was checked for good with the Hosmer-Lemeshow goodness-of-fit test. Finally, from multivariable analysis, a *p*-value less than 0.05 was considered statistically significant, and the AOR with 95% CI was used to determine the strength of association.

## Results

### Socio-demographic characteristics of the respondents

A total of 510 participants were involved in this study, making the overall response rate of 94%.

The mean age of the respondents was 29.26 years [standard deviation (SD) ± 3.95] with a range of 22–46 years. Among the study participants, more than half (56.7%) were males, and 262 (51.4%) were single. The majority of participants (84.5%) were degree holders. Of the respondents (19.8%) were working in the surgical unit and more than half of the respondents (52.2%) were less than 5 years of experience. Similarly, 305 (59.8%) of the participant’s monthly income was between 4,185 and 6,520 ETB (1 USD = 44.03 ETB, during the data collection period) (Table 1).

**TABLE 1:** Socio-demographic characteristics of nurses working at comprehensive specialized hospitals in Northwest Ethiopia, 2021 (*n* = 510).

Variables	Categories	Frequency	Percent (%)
Age	<25 years	38	7.5
	25–30 years	321	62.9
	31–35 years	113	22.2
	>35 years	38	7.5
Sex	Male	289	56.7
	Female	221	43.3
Marital status	Married	223	43.7
	Single	262	51.4
	Others*	25	4.9
Educational status	Diploma	39	7.6
	BSc degree	431	84.5
	MSc and above	40	7.8
Working unit	Internal medicine	98	19.2
	Surgery	101	19.8
	Gynecology and obstetrics	59	11.6
	Emergency	75	14.7
	ICU	58	11.4
	Psychiatry	7	1.4
	Pediatrics	75	14.7
	OPD	40	7.8
Work experience	<5 years	266	52.2
	5–10 years	215	42.2
	>10 years	29	5.7
Monthly income	<4,185	43	8.4
	4,185–6,520	305	59.8
	>6,520	162	31.8

*Others: Divorced, widowed, and separated.

### Psychological, clinical, and shift work sleep disorder characteristics of the respondents

The psychological characteristics indicated that out of the total study participants, 183 (35.9%), 207 (40.6%), and 169 (33.1%), were found to have the symptoms of depression, anxiety, and stress respectively. Among the participants, 70 (13.7%) nurses reported chronic medical illness and more than one-third (36.5%) had shift work sleep disorder (Table 2).

**TABLE 2:** Psychological, clinical, and shift work sleep disorder characteristics of nurses working at comprehensive specialized hospitals in Northwest Ethiopia, 2021 (*n* = 510).

Variables	Categories	Frequency (*n* = 510)	Percent (%)
Depressive symptoms	Yes	183	35.9
	No	327	64.1
Anxiety symptoms	Yes	207	40.6
	No	303	59.4
Stress	Yes	169	33.1
	No	341	66.9
Chronic medical illness	Yes	70	13.7
	No	440	86.3
Shift work sleep disorder	Yes	186	36.5
	No	324	63.5

### Description of substance use and psychosocial characteristics of participants

About one-third (33.1%) of participants were current alcohol users, whereas 49 (9.6%) and 43 (8.4%) were current khat users and current cigarette smokers, respectively. Regarding social support, the majority (36.9%) had received moderate social support (Table 3).

**TABLE 3:** Description of substance use and psychosocial characteristics of nurses working at comprehensive specialized hospitals in Northwest Ethiopia, 2021 (*n* = 510).

Variables	Categories	Frequency	Percentage (%)
Ever use of khat	Yes	68	13.3
	No	442	86.7
Current use of khat	Yes	49	9.6
	No	461	90.4
Ever use of alcohol	Yes	180	35.3
	No	330	64.7
Current use of alcohol	Yes	169	33.1
	No	341	66.9
Ever use of cigarette	Yes	52	10.2
	No	458	89.8
Current use of cigarette	Yes	43	8.4
	No	467	91.6
Ever use of shisha	Yes	11	2.2
	No	499	97.8
Current use of shisha	Yes	8	1.6
	No	502	98.4
Social support	Low	168	32.9
	Moderate	188	36.9
	Strong	154	30.2

### Prevalence of sleep quality among respondents

In the current study, the prevalence of poor sleep quality among nurses was 75.5% [95% CI (71.8, 79.1)]. The global PSQI score (computed using the component scores) ranged from 0 to 16 with a mean of 7.21 (±1.42). About 228 (44.7%) of respondents stated that their subjective experience of sleep quality was fairly good. Regarding the sleep latency, the number of minutes usually taken to fall asleep each night, 219 (42.9%) of the participants were 16–30 min. About 48 (9.4%) of the respondent’s sleep duration that hours of sleep per night were less than 5 h (Table 4).

**TABLE 4:** Sleep quality and its component score among nurses working at comprehensive specialized hospitals in Northwest Ethiopia, 2021 (*n* = 510).

Components	Score	Frequency	Percentage (%)
Subjective sleep quality	Very good	140	27.5
	Fairly good	228	44.7
	Fairly bad	125	24.5
	Very bad	17	3.3
Sleep latency	0	53	10.4
	1–2	186	36.5
	3–4	198	38.8
	5–6	73	14.3
Minutes that take to fall sleep	≤15 min	99	19.4
	16–30 min	219	42.9
	31–60 min	192	37.5
	>60 min	1	0.2
Sleep duration	Greater than 7 h	212	41.6
	6–7 h	153	30.0
	5–6 h	97	19.0
	Less than 5 h	48	9.4
Habitual sleep efficiency*	Greater than 85%	254	49.8
	75–85%	114	22.4
	65–74%	74	14.5
	Less than 65%	68	13.3
Sleep disturbance	0	46	9.0
	1–9	271	53.1
	10–18	192	37.6
	19–27	1	0.2
Use of sleep medication	Not during the past month	435	85.3
	Less than once a week	45	8.8
	Once or twice a week	27	5.3
	Three or more times each week	3	0.6
Daytime dysfunction	0	136	26.7
	1–2	162	31.8
	3–4	172	33.7
	5–6	40	7.8
Global poor sleep quality	Yes	385	75.5
	No	125	24.5

### Factors associated with poor sleep quality among nurses

In the bivariate analysis, sex, SWSD, current alcohol drinkers, current cigarette smoking, having a chronic medical illness, depressive symptoms, anxiety symptoms, stress, and social support showed a *p*-value of <0.25 and became a candidate for multivariable analysis. In multivariable binary logistic regression variables; sex, current alcohol use, symptoms of depression, anxiety, and stress were significantly associated with poor sleep quality at a *p*-value less than 0.05.

The odds of poor sleep quality among female participants were 1.72 times higher as compared to males (AOR = 1.72, 95% CI: 1.19, 2.28). Those participants with current alcohol drinkers were nearly two times more likely to have poor sleep quality than those non-current alcohol drinkers (AOR = 1.84, 95% CI: 1.27, 3.13). Those participants who had depressive symptoms were two times more likely to have poor sleep quality than participants who had no depressive symptoms (AOR = 2.24, 95% CI: 1.24, 3.85). Similarly, those nurses with anxiety symptoms were 2.12 times more likely to have poor sleep quality than their counterparts (AOR = 2.12, 95% CI: 1.23, 3.62). Furthermore, the odds of having poor sleep quality among participants who had stress were about 2.85 times higher as compared with the referent groups (AOR = 2.85, 95% CI: 1.67–4.82) (Table 5).

**TABLE 5:** Bi-variables and multi-variables regression analysis between poor sleep quality and explanatory variables among nurses working at comprehensive specialized hospitals in Northwest Ethiopia, 2021.

Variables	Categories	Poor sleep quality		COR (95% CI)	AOR (95% CI)	*P*-value
		Yes	No			
Sex	Female	174	47	1.47 (0.91–2.07)	1.72 (1.19–2.28)*	0.020*
	Male	211	78	1.00	1.00	
Shift work sleep disorder	Yes	146	40	1.39 (0.85–1.99)	1.39 (0.69–1.75)	0.696
	No	239	85	1.00	1.00	
Current alcohol drink	Yes	141	28	2.00 (1.25–3.29)	1.84 (1.27–3.13)*	0.024*
	No	244	97	1.00	1.00	
Current cigarette smoking	Yes	37	6	2.11 (0.87–5.12)	1.52 (0.58–4.00)	0.396
	No	348	119	1.00	1.00	
Depressive symptoms	Yes	162	21	3.69 (2.26–5.99)	2.24 (1.24–3.85)*	0.01*
	No	223	104	1.00	1.00	
Anxiety	Yes	180	27	3.19 (1.99–5.10)	2.12 (1.23–3.62)*	0.006*
	No	205	95	1.00	1.00	
Stress	Yes	150	19	3.56 (2.19–6.05)	2.85 (1.67–4.82)*	<0.001*
	No	235	106	1.00	1.00	
Chronic medical illness	Yes	57	13	1.59 (0.79–2.84)	1.21 (0.60–2.41)	0.605
	No	328	112	1.00	1.00	
Social support	Low	134	34	1.68 (1.01–2.89)	1.04 (0.60–1.78)	0.903
	Moderate	143	45	1.35 (.84–2.19)	0.82 (0.82–1.44)	0.475
	High	108	46	1.00	1.00	

N.B. 1.00 = reference, * statistically significant at *P*-value < 0.05, Hosmer-Lemeshow test = 0.56, AOR, Adjusted odds Ratio, COR, Crude odds Ratio, Maximum VIF = 1.39.

## Discussion

In this study, the prevalence of poor sleep quality among nurses working at comprehensive specialized hospitals in Northwest Ethiopia was 75.5% (95% CI: 71.8–79.1). The current study is consistent with the studies done in UK 78% (14), China 76.3% (16), and Nigeria 77.1% (18). However, the proportion of poor sleep quality was higher than in a study conducted in the United States 66% (13), South India 46.3% (33), Turkey 61.9% (23), Taiwan 68.9% (34), China 63.9% (20), Italia 54.6% (15), and Jimma, Ethiopia 70.6% (19). This difference might be due to differences in human power, adequate facility, and the health working system compared to this study. For example, most of the previous studies were conducted in developed countries. Nurses in developed countries may have better and safer working conditions and adequate facilities than nurses in developing countries. This can be due to greater economic resources and regulatory policies that support quality occupational health and safety precautions in most developed countries. In developing countries like Ethiopia, they have a low nurse-patient ratio and limited qualified human resources compared to a disease burden that increases workload and stressors which increase the poor sleep quality (35, 36). Another possible reason for the higher prevalence of poor sleep quality in this study compared to Jimma (19) might be related to concerns of the contagious nature of the COVID-19 outbreak whereas, the previous study was done before the outbreak. Nurses are the frontline health professionals for the outbreak of COVID-19 is a serious challenge for them. They are at constant risk of contracting the virus and be infected with the disease from their patients. They are also under great stress of keeping themselves and their family healthy from the virus. These may all result in poor sleep quality and short sleep duration. This finding is supported by a recent study done in China (37).

On the other hand, the prevalence of poor sleep quality in this study was lower than in studies done in Iran 85.7% (21) and in Korea 79.8% (17). The possible reason for the difference might be because nurses included in those two studies were only shift working nurses, whereas, the current study included all nurses regardless of their shift working status. Shiftwork is strongly linked with shiftwork sleep disorders which also compromises sleep quality (21). The other variation might be due to the PSQI score cut point difference, the current study used PSQI >5 while the previous study in Iran (21) and Korea (17) was a PSQI score ≥5.

Regarding, factors affecting poor sleep quality, female nurses were nearly two times more likely to have poor sleep quality as compared to male participants. Different studies reported that poor sleep quality was more frequent in female nurses than in males (20–22). This finding might be related to the female physiological and psychological features. Female nurses need to work and take care of their families at the same time, such that their life pressure is higher than that of male nurses. This could be also that females have more responsibilities for looking after the home and children, and the majority of housework, which may aggravate sleep problems and tiredness (38). Sleep disturbances are frequently symptoms of anxiety and depression, which may be reflected in the sleep quality difference caused by gender differences (32). Thus, their sleep quality might be affected more easily.

The current study also showed participants who had depressive symptoms were two times more likely to have poor sleep quality than undepressed participants. Similar to a finding of different studies from Taiwan (25) and Jordan (24). The possible reason might be medical emergencies added to the tension of patient care, dealing with grief and loss when a patient dies these conditions might increase a nurse’s stress level or depressive symptoms (24). Additionally, it was discovered that poor sleep quality was linked to depressive symptoms because depressive symptoms lower the number of serotonin neurotransmitters in the brain of a depressed individual, which results in impaired cognitive performance (39). It also indicated that the same neurotransmitter systems that regulate mood, interest, energy, and other function may be disturbed in depressive symptoms and also regulate sleep. Serotonergic neurons play a critical role in the modulation of the onset and maintenance of sleep. So, depressive symptoms are associated with poor sleep quality caused by the dysfunction of serotonergic systems (40).

In the current study, we found that participants who had anxiety symptoms were 2 times more likely to have poor sleep quality than participants who had no anxiety symptoms. The finding was in line with studies done in Taiwan (25) and Jordan (24). The probable reason might be nurses often need to deal with patients with critical and severe diseases or with complex diseases, which may lead to rapid working rhythms and high mental tension and anxiety symptoms that are associated with sleep quality (41). This results, in nurses, may be easier to suffer from a lack of sleep. Additionally, nurses usually experience unpleasant emotions and anxiety symptoms from their work due to the fact of the fast work pace and many emergencies that might contribute largely to the development of poor sleep among nurses (41).

This finding also revealed that respondents who had stress were nearly three times more likely to have poor sleep quality than their counterparts. This was in line with the studies done in China (20), Korea (17), and Ethiopia (19). Nurses under a persistently high state of stress may feel burned out, frustrated, irritated, and exhausted they may have poor sleep quality (42). The other possible reason for this might be medical emergencies add to the tension of patient care, and nurses deal with grief and loss when a patient dies increasing their stress that disturbing their sleep quality. Another possible justification might be that physiologically, sleep and stress are closely linked to the hypothalamus- pituitary adrenal (HPA) axis which may explain the close relationship between these two variables (43). Stress is accompanied by a decrease in slow-wave, rapid eye movement, and increased sleep deprivation (43).

In addition, those nurses who use current alcohol were nearly two times more likely to experience poor sleep quality. This result was agreed to by a previous study (44). The reasons underlying these associations could be related to drinking might directly affect sleep structure and sleep quality by inhibiting rapid eye movement sleep and increasing slow-wave sleep in the first half of sleep (45). The other possible justification for this association might be due to ethanol acting as a central nervous system depressant, which might contribute to its impact on sleep (46). This might be also because drinking alcohol is common among stressed-out nurses as a coping strategy that may harm their health in consequence they turn to drink as a stress-reliever and to help to overcome the stress that affects their sleep (47).

## Limitation of the study

The limitation of this study might be due to the nature of the study design, which cannot establish a temporal relationship between outcome and independent variables. The use of a self-administered questionnaire that relied on subjective measures of sleep quality and other variables may have the possibility of recall bias; some people may have over or underreported.

## Conclusion

This study showed that poor sleep quality was common among nurses accounting for about three fourth of them. The distribution of poor sleep quality among nurses showed that it was higher in females, current alcohol drinkers, in nurses who had symptoms of depression, anxiety and stress. Therefore, the sleep quality of nurses needs to be cautiously explored and due attention has to be given to designing intervention programs aimed at addressing the identified risk factors. To expand on the current findings, additional research on the risk factors for poor sleep quality utilizing multiple study designs should be done.

## Data availability statement

The original contributions presented in the study are included in the article/supplementary material, further inquiries can be directed to the corresponding author.

## Ethics statement

The studies involving human participants were reviewed and approved by University of Gondar Ethical Institutional Review Board. The patients/participants provided their written informed consent to participate in this study.

## Author contributions

TS, HK, and TA conceived the idea for the study, wrote the proposal, participated in data collection, and did the analysis. FG, BT, and GN approved the proposal with some revisions and revised subsequent drafts of the manuscript. TA prepared the manuscript for publication. All authors read and approved the final manuscript.

## Abbreviations

AOR: Adjusted Odds Ratio
BSc: Bachelor of Science
CI: Confidence Interval
CMHs: Comprehensive Specialized Hospitals
COR: Crude Odd Ratio
COVID-19: Corona Virus disease 2019
DASS-21: Depression Anxiety Stress Scales
ETB: Ethiopian Birr
GC: Gregorian calendar
MSc: Master of Science
OR: Odds Ratio
PSQI: Pittsburgh Sleep Quality Index
SPSS: Statically Package of Social Science
SWSD: Shift Work Sleep Disorder
UK: United Kingdom
UOG: University of Gondar
USA: the United States of America
WHO: World Health Organization.

## References

[1] American Psychiatric Association. *Diagnostic and Statistical Manual of Mental Disorders.* Arlington, VA: American Psychiatric Publishing, Inc (1995).

[2] Zielinski MR McKenna JT McCarley RW. Functions and mechanisms of sleep. *AIMS Neurosci.* (2016) 3:67. 10.3934/Neuroscience.2016.1.67 28413828 PMC5390528

[3] Tang J Liao Y Kelly BC Xie L Xiang Y-T Qi C Gender and regional differences in sleep quality and insomnia: a general population-based study in Hunan Province of China. *Sci Rep.* (2017) 7:1–9. 10.1038/srep43690 28262807 PMC5337959

[4] Lemola S Räikkönen K Scheier MF Matthews KA Pesonen AK Heinonen K Sleep quantity, quality and optimism in children. *J Sleep Res.* (2011) 20(1 Pt. 1):12–20. 10.1111/j.1365-2869.2010.00856.x 20561178 PMC4160149

[5] Shi D Zhao J Liu X. Investigation and analysis of current stress status of nurses and countermeasures. *Chin J Pract Nurs.* (2011) 27:4–7.

[6] Caruso CC. Negative impacts of shiftwork and long work hours. *Rehabil Nurs.* (2014) 39:16–25. 10.1002/rnj.107 23780784 PMC4629843

[7] Wells ME Vaughn BV. Poor sleep challenging the health of a nation. *Neurodiagn J.* (2012) 52:233–49. 23019761

[8] Centers for Disease Control and Prevention. *Insufficient Sleep is a Public Health Problem.* Atlanta, GA: US Department of Health and Human Services (2015).

[9] Shao MF Chou YC Yeh MY Tzeng WC. Sleep quality and quality of life in female shift−working nurses. *J Adv Nurs.* (2010) 66:1565–72. 10.1111/j.1365-2648.2010.05300.x 20492021

[10] Åkerstedt T. Shift work and disturbed sleep/wakefulness. *Occup Med.* (2003) 53:89–94. 10.1093/occmed/kqg046 12637592

[11] Çelik S Taşdemir N Kurt A Ýlgezdi E Kubalas Ö. Fatigue in intensive care nurses and related factors. *Int J Occup Environ Med.* (2017) 8:199. 10.15171/ijoem.2017.1137 28970594 PMC6679605

[12] Zeng L-N Yang Y Wang C Li X-H Xiang Y-F Hall BJ Prevalence of poor sleep quality in nursing staff: a meta-analysis of observational studies. *Behav Sleep Med.* (2020) 18:746–59. 10.1080/15402002.2019.1677233 31672062

[13] Beebe D Chang JJ Kress K Mattfeldt−Beman M. Diet quality and sleep quality among day and night shift nurses. *J Nurs Manag.* (2017) 25:549–57. 10.1111/jonm.12492 28695685

[14] McDowall K Murphy E Anderson K. The impact of shift work on sleep quality among nurses. *Occup Med.* (2017) 67:621–5. 10.1093/occmed/kqx152 29040745

[15] Di Muzio M Diella G Di Simone E Novelli L Alfonsi V Scarpelli S Nurses and night shifts: poor sleep quality exacerbates psychomotor performance. *Front Neurosci.* (2020) 14:1050. 10.3389/fnins.2020.579938 33154716 PMC7591770

[16] Dong H Zhang Q Zhu C Lv Q. Sleep quality of nurses in the emergency department of public hospitals in China and its influencing factors: a cross-sectional study. *Health Qual Life Outcomes.* (2020) 18:1–9. 10.1186/s12955-020-01374-4 32349759 PMC7191763

[17] Park E Lee HY Park CSY. Association between sleep quality and nurse productivity among Korean clinical nurses. *J Nurs Manag.* (2018) 26:1051–8. 10.1111/jonm.12634 29855101

[18] Kolo E Ahmed A Hamisu A Ajiya A Akhiwu B. Sleep health of healthcare workers in Kano, Nigeria. *Niger J Clin Pract.* (2017) 20:479–83. 28406131 10.4103/1119-3077.204378

[19] Olana DD Ayana AM Abebe ST. Sleep quality and its associated factors among nurses in jimma zone public hospitals. Southwest Ethiopia, 2018. *Sleep Hypnosis.* (2019) 21:271–80.

[20] Dong H Zhang Q Sun Z Sang F Xu Y. Sleep disturbances among Chinese clinical nurses in general hospitals and its influencing factors. *BMC Psychiatry.* (2017) 17:1–9. 10.1186/s12888-017-1402-3 28673267 PMC5496307

[21] Akbari V Hajian A Mirhashemi M. Evaluating of sleep quality in shift-work nurses. *Iran J Sleep Disored Ther.* (2016) 5:225. 10.4172/2167-0277.1000225

[22] Korompeli A Chara T Chrysoula L Sourtzi P. Sleep disturbance in nursing personnel working shifts. *Nurs Forum.* (2013) 48:45–53. 10.1111/nuf.12005 23379395

[23] Tarhan M Aydın A Ersoy E Dalar L. The sleep quality of nurses and its influencing factors. *Eur J Pulmonol.* (2018) 2:78–84.

[24] AbuRuz ME Hayeah HMA. Insomnia induced by night shift work is associated with anxiety, depression, and fatigue, among critical care nurses. *Adv Stud Biol.* (2017) 9:137–56. 10.12988/asb.2017.738

[25] Hsieh ML Li YM Chang ET Lai HL Wang WH Wang SC. Sleep disorder in Taiwanese nurses: a random sample survey. *Nurs Health Sci.* (2011) 13:468–74. 10.1111/j.1442-2018.2011.00641.x 22011090

[26] Liu Y Chen G. Nurse’s sleep quality in a three level hospital in Guangzhou. *China J Health Psychol.* (2015) 237:989–92.

[27] Buysse DJ Reynolds IIICF Monk TH Berman SR Kupfer DJ. The Pittsburgh sleep quality index: a new instrument for psychiatric practice and research. *Psychiatry Res.* (1989) 28:193–213. 10.1016/0165-1781(89)90047-4 2748771

[28] Salahuddin M Maru TT Kumalo A Pandi-Perumal SR Bahammam AS Manzar MD. Validation of the Pittsburgh sleep quality index in community dwelling Ethiopian adults. *Health Qual Life Outcomes.* (2017) 15:1–7. 10.1186/s12955-017-0637-5 28347341 PMC5369003

[29] American Academy of Sleep Medicine. *International Classification of Sleep Disorders.* Westchester, Ill: American Academy of Sleep Medicine (2005).

[30] Henry JD Crawford JR. The short−form version of the Depression Anxiety Stress Scales (DASS−21): construct validity and normative data in a large non−clinical sample. *Br J Clin Psychol.* (2005) 44:227–39. 10.1348/014466505X29657 16004657

[31] Group WAW. The alcohol, smoking and substance involvement screening test (ASSIST): development, reliability and feasibility. *Addiction.* (2002) 97:1183–94. 10.1046/j.1360-0443.2002.00185.x 12199834

[32] Dalgard OS Dowrick C Lehtinen V Vazquez-Barquero JL Casey P Wilkinson G Negative life events, social support and gender difference in depression. *Soc Psychiatry Psychiatr Epidemiol.* (2006) 41:444–51. 10.1007/s00127-006-0051-5 16572275

[33] Khade Y Behera S Korradi S. Study of insomnia, day time sleepiness and sleep quality among South Indian Nurses. *J Clin Diagn Res.* (2018) 12:9–12. 10.7860/JCDR/2018/32602.11392

[34] Chueh K-H Chen K-R Lin Y-H. Psychological distress and sleep disturbance among female nurses: anxiety or depression? *J Transcult Nurs.* (2019) 32:14–20. 10.1177/1043659619881491 31625463

[35] Verma A Kishore J Gusain S. A comparative study of shift work effects and injuries among nurses working in rotating night and day shifts in a tertiary care hospital of North India. *Iran J Nurs Midwifery Res.* (2018) 23:51. 10.4103/ijnmr.IJNMR_15_17 29344047 PMC5769186

[36] Ilhan MN Durukan E Aras E Türkçüoðlu S Aygün R. Long working hours increase the risk of sharp and needlestick injury in nurses: the need for new policy implication. *J Adv Nurs.* (2006) 56:563–8. 10.1111/j.1365-2648.2006.04041.x 17078831

[37] Xiao H Zhang Y Kong D Li S Yang N. The effects of social support on sleep quality of medical staff treating patients with coronavirus disease 2019 (COVID-19) in January and February 2020 in China. *Med Sci Monit.* (2020) 26:e923549. 10.12659/MSM.923549 32132521 PMC7075079

[38] Pati AK Chandrawanshi A Reinberg A. Shift work: consequences and management. *Curr Sci.* (2001):32–52. 10.1076/brhm.32.1.45.7286

[39] Taylor DJ. Insomnia and depression. *Sleep.* (2008) 31:447. 10.1093/sleep/31.4.447 18457230 PMC2279745

[40] Meerlo P Sgoifo A Suchecki D. Restricted and disrupted sleep: effects on autonomic function, neuroendocrine stress systems and stress responsivity. *Sleep Med Rev.* (2008) 12:197–210. 10.1016/j.smrv.2007.07.007 18222099

[41] Zhang Q Dong H Zhu C Liu G. Low back pain in emergency ambulance workers in tertiary hospitals in China and its risk factors among ambulance nurses: a cross-sectional study. *BMJ Open.* (2019) 9:e029264. 10.1136/bmjopen-2019-029264 31537564 PMC6756463

[42] Saini R Kaur S Das K. Assessment of stress and burnout among intensive care nurses at a tertiary care hospital. *J Mental Health Hum Behav.* (2011) 16:43–8.

[43] Kashani M Eliasson A Vernalis M. Perceived stress correlates with disturbed sleep: a link connecting stress and cardiovascular disease. *Stress.* (2012) 15:45–51. 10.3109/10253890.2011.578266 21682656

[44] Manzar MD Salahuddin M Alamri M Maru TT Pandi-Perumal SR Bahammam AS. Poor sleep in concurrent users of alcohol, khat, and tobacco smoking in community-dwelling Ethiopian adults. *Ann Thoracic Med.* (2018) 13:220–5. 10.4103/atm.ATM_36_18 PMC6196670 30416593

[45] Garcia AN Salloum IM. Polysomnographic sleep disturbances in nicotine, caffeine, alcohol, cocaine, opioid, and cannabis use: a focused review. *Am J Addict.* (2015) 24:590–8. 10.1111/ajad.12291 26346395

[46] Erdozain AM Morentin B Bedford L King E Tooth D Brewer C Alcohol-related brain damage in humans. *PLoS One.* (2014) 9:e93586. 10.1371/journal.pone.0093586 24699688 PMC3974765

[47] Hope A Kelleher CC O’connor M. Lifestyle practices and the health promoting environment of hospital nurses. *J Adv Nurs.* (1998) 28:438–47. 10.1046/j.1365-2648.1998.00791.x 9725743

